# Food-Grade Metal Oxide Nanoparticles Exposure Alters Intestinal Microbial Populations, Brush Border Membrane Functionality and Morphology, In Vivo (*Gallus gallus*)

**DOI:** 10.3390/antiox12020431

**Published:** 2023-02-09

**Authors:** Jacquelyn Cheng, Nikolai Kolba, Alba García-Rodríguez, Cláudia N. H. Marques, Gretchen J. Mahler, Elad Tako

**Affiliations:** 1 Department of Food Science, Cornell University, 411 Tower Road, Ithaca, NY 14853, USA; 2 Department of Biomedical Engineering, Binghamton University, 4400 Vestal Parkway East, Binghamton, NY 13902, USA; 3 Department of Biological Sciences, Binghamton University, 4400 Vestal Parkway East, Binghamton, NY 13902, USA

**Keywords:** ingestion, nanotoxicity, microbiota, iron, brush border membrane

## Abstract

Among food additive metal oxide nanoparticles (NP), titanium dioxide (TiO₂) and silicon dioxide (SiO₂) are commonly used as food coloring or anti-caking agents, while zinc oxide (ZnO) and iron oxide (Fe₂O₃) are added as antimicrobials and coloring agents, respectively, and can be used as micronutrient supplements. To elucidate potential perturbations associated with NP consumption on gastrointestinal health and development, this in vivo study utilized the *Gallus gallus* (broiler chicken) intraamniotic administration to assess the effects of physiologically relevant concentrations of food-grade metal oxide NP on brush border membrane (BBM) functionality, intestinal morphology and intestinal microbial populations in vivo. Six groups with 1 mL injection of the following treatments were utilized: non-injected, 18 MΩ DI H_2_O; 1.4 × 10^−6^ mg TiO_2_ NP/mL, 2.0 × 10^−5^ mg SiO_2_ NP/mL, 9.7 × 10^−6^ mg ZnO NP/mL, and 3.8 × 10^−4^ mg Fe_2_O_3_ NP/mL (*n* = 10 per group). Upon hatch, blood, cecum, and duodenum were collected to assess mineral (iron and zinc) metabolism, BBM functional, and pro-inflammatory-related protein gene expression, BBM morphometric analysis, and the relative abundance of intestinal microflora. Food additive NP altered mineral transporter, BBM functionality, and pro-inflammatory cytokine gene expression, affected intestinal BBM development and led to compositional shifts in intestinal bacterial populations. Our results suggest that food-grade TiO₂ and SiO₂ NP have the potential to negatively affect intestinal functionality; food-grade ZnO NP exposure effects were associated with supporting intestinal development or compensatory mechanisms due to intestinal damage, and food-grade Fe₂O₃ NP was found to be a possible option for iron fortification, though with potential alterations in intestinal functionality and health.

## 1. Introduction

Nanoparticles (NP) are commonly ingested due to their widespread usage in the food and agriculture industries, and their impact on gastrointestinal (GI) development is not well understood [1,2,3,4]. Within the food industry, NP are utilized due to their unique properties in enhancing food texture, flavor, color, consistency, stability, and preservation (antibacterial activity). Further, NP have been utilized to enhance nutrient bioavailability, where Fe_2_O_3_ and ZnO NP have been found to have higher relative bioavailability, faster dissociation, and minimal effects on organoleptic properties when compared with conventional fortificants [5,6]. NP are also found in food packaging applications to improve packaging flexibility and gas barrier properties and thus can migrate into the food matrix [7,8,9]. In agriculture, NP are used for developing novel agrochemicals (i.e., fertilizers and pesticides) and sensors for the identification of nutrients or contaminants within food products or water [10,11]. Numerous studies have previously demonstrated food-grade metal oxides in the nanometer range and detected their presence in many food consumer products [12,13,14,15]. As human exposure to NP through food, water, and the environment is inevitable, it is key to understand the effects of NP on GI health and development.

Following ingestion, NP interact with the GI tract, where GI tract biological features and NP physicochemical properties can impact the effects of NP on GI health and development [3,7]. The pH varies within different compartments of the GI tract, which can affect NP aggregation and surface chemistry [16]. In addition to serving as the key site of nutrient digestion and absorption, the small intestine also serves as protective secretory and immune functions [17]. The mucus layer, which is produced by goblet cells and is comprised of mucopolysaccharides and glycoproteins, provides a physical barrier to luminal bacteria and concentrates antibacterial peptides and proteins secreted from Paneth cells and enterocytes near the epithelium [17,18]. Additionally, this mucin houses intestinal microbiota, which contributes to the digestion of minerals and interacts with epithelial cells to maintain an effective gut barrier [19,20,21]. Potential outcomes of NP ingestion include absorption, by which NP can gain access to blood and thus to other organs, deposition on mucosa, and gut microbiota modulation, which have critical roles in maintaining normal gut physiologic, metabolic, and immune functions [22,23]. Accordingly, the consequences of NP ingestion from food sources on GI functions require further scrutiny, and the evaluation of NP must consider the effects of this perturbation on microbiota composition, intestinal functionality, and inflammatory status.

Many food additive NP, such as TiO_2_, SiO_2_, ZnO, and Fe_2_O_3_ NP, have been reported to differentially behave and interact with microorganisms, mainly due to their size-related physicochemical properties, agglomeration, and aggregation level, as well as their dissolution rate (ion release) [24,25,26]. Although each NP presents different molecular compositions, similar toxicological strategies have been hypothesized and described according to each NP type. Reactive oxygen species (ROS)-mediated toxicity is believed to be the primary mechanism of in vitro NP cytotoxicity, including TiO_2_, SiO_2_, ZnO, and Fe_2_O_3_ NP, and in some cases, has also been reported to be associated with DNA damage and genotoxicity [27,28,29]. However, the disturbance of cell membrane integrity due to mechanical interactions between NP clusters may be another major mechanism of TiO_2_ and SiO_2_ cytotoxicity where these types of food-grade NP altered the distribution of tight junction proteins due to oxidative stress-induced changes [30,31,32]. ZnO and Fe_2_O_3_ NP cytotoxic mechanisms have also been closely related to their dissolution in the media. ZnO and Fe_2_O_3_ are amphoteric molecules, and as such, can behave differentially depending on the pH of the solution and are more prone to the release of ions in acidic solutions [7]. Several studies have proposed the release of Zn^2+^ in the media as a key cytotoxic and antibacterial mechanism for ZnO NP, which has a significant effect on amino acid metabolism, enzyme system disruption, and inactive transport inhibition [33].

Our previous work with an in vitro model of the intestinal epithelium has shown that exposure to TiO_2_, SiO_2_, ZnO, and Fe_2_O_3_ NP changes the functionality of intestinal epithelial cells and causes inflammation [7,14,34,35,36]. TiO_2_ NP have been shown to negatively impact the mucus layer by decreasing mucin thickness and increasing neutral and acidic mucins when in the presence of *Escherichia coli* and *Lacticaseibacillus rhamnosus,* leading to potential pathological conditions [36]. Guo et al. (2017) demonstrated that TiO_2_ NP could significantly alter brush border membrane (BBM) enzyme functionality, iron (Fe) and zinc (Zn) nutrient transport, intestinal alkaline phosphatase activity, and tight junction functionality [35]. SiO_2_ NP have been shown in vitro to increase glucose transportation, decrease intestinal nutrient absorption, significantly alter the gene expression levels of nutrient transport proteins, and induce ROS and pro-inflammatory signaling [34]. ZnO NP were shown to likely dissolve during digestion in vitro, though it was found that ZnO NP negatively affected glucose absorption and mineral absorption by altering the cell microvilli surface [14]. Further, our previous in vitro study found that BBM enzyme activity was modulated following exposure to in vitro digested TiO_2_, SiO_2_, ZnO, and Fe_2_O_3_ NP, and in the presence of naturally present gut microbiota, *E. coli* or *L. rhamnosus*, some of the BBM enzyme activities that were affected by food additive NP exposure were ameliorated due to bacterial adsorption of metal oxide NP [7].

In the well-established in vivo *Gallus gallus* model [37,38,39], we previously demonstrated that physiologically relevant doses of chemical-grade TiO_2_ and SiO_2_ NP have the potential to negatively affect intestinal functionality and health, which is congruent with previous in vitro study findings [40]. The *G. gallus* model has previously been utilized to assess mineral bioavailability due to its sensitivity to dietary mineral content manipulation and thus can serve as a model for human dietary mineral bioavailability and absorption [38,39,41,42]. Further, there is >85% gene homology in intestinal BBM proteins involved in mineral transport between humans and *G. gallus* [43]. Finally, the *G. gallus* has a complex gut microbiome, significantly and directly influenced by host genetics, the environment, and diet, with a significant resemblance in the gut microbiota of *G. gallus* and humans at the phylum level [20,44,45].

Our present in vivo study assessed the impact of food-grade TiO_2_, SiO_2_, ZnO, and Fe_2_O_3_ NP at human-relevant dosages on intestinal health, function, and microbial populations. Due to widespread exposure resulting from human consumption of food additive NP, this present study utilized food-grade NP to study the impact of NP exposure on GI functionality. This study is a follow-up to our previous in vivo study, where we assessed the effects of chemical-grade TiO_2_, SiO_2_, and ZnO NP on gut health and functionality in vivo using the embryonic stage of the *G. gallus* [40]. Based on our previous in vitro results where the physiologically relevant doses of TiO_2_, SiO_2_, ZnO, and Fe_2_O_3_ NP were found to alter the activity of most of the tested digestive enzymes in a non-toxic and non-concentration-dependent way, we focused our attention on only human-relevant dose exposures [7,14,34,35]. This study utilized the intraamniotic administration approach to assess and screen the effects of food-grade TiO_2_, SiO_2_, ZnO, and Fe_2_O_3_ NP at human-relevant dosages on BBM functionality through evaluating duodenal gene expression of biomarkers of mineral status, BBM digestive and absorptive ability, immune function, and inflammation in vivo in the *G. gallus*. A secondary objective was to evaluate the effects of these food-grade metal oxide NP on cecal bacterial populations by quantifying the relative abundances of health-promoting populations (*Bifidobacterium* spp. and *Lactobacillus* spp.) versus those of potentially pathogenic bacteria (*E. coli* and *Clostridium* spp.). We hypothesize that food-grade NP will have a negative effect on BBM functionality and development and alter populations of intestinal bacteria.

## 2. Materials and Methods

### 2.1. Sonicator Calibration and Critical Delivered Sonication Energy Determination

A BRANSON Sonifier^®^ SFX550 (Branson Ultrasonics, Danbury, CN, USA) was used to facilitate the disruption of NP agglomerates by applying ultrasonic energy. Since two different instruments operating at the same level or amplitude (%) setting can deliver significantly different effective acoustic powers to the same suspension, equipment calibration is needed [46]. Sonication equipment calibration was performed through a calorimetric method to ensure accurate power application and reporting of delivered sonication energy (DSE) (see Supplementary Materials for detailed methodology). Therefore, the final operating settings chosen to disperse our NP were: 10% of amplitude for 2 min, where the delivered power is 7.33 watts, and the delivered sonication energy is 87.9 Joules/mL.

### 2.2. Food-Grade Nanoparticles Preparation and Dosimetry

Food-grade TiO_2_ (Fiorio Colori, AromataGroup, Milan, Italy); SiO_2_ (Spectrum Chemical Mfg. Corp., New Brunswick, NJ, USA); ZnO (Spectrum Chemical Mfg. Corp., New Brunswick, NJ, USA); and Fe_2_O_3_ (Town End, Leeds, UK) were used in this study. These additives were previously characterized by Garcia-Rodriguez et al. [7]. NP formulations were prepared and dispersed based on protocols established by the OECD (Organization for Economic Cooperation and Development) and NIST (National Institute of Standards and Technology) [46,47]. Briefly, in a 20 mL scintillation vial, 10 mg of NP powder was dissolved in 10 mL of sterile 18 MΩ DI H_2_O and sonicated for 2 min at 10% amplitude in a continuous mode. The probe sonicator equipped with a disruptor horn of ½” diameter (BRANSON Sonifier^®^ SFX550, Emerson Electric Co., St. Louis, MO, USA) was fully immersed in the NP suspension without touching the scintillation vial. To avoid heating the samples, the scintillation vial containing 1 mg/mL of each NP was placed in an ice bath while sonicating. The disruptor horn was sterilized before and after NP preparation by sonicating a solution of 50% ethanol for 5 min. Physiologically relevant NP concentrations were chosen for this study based on previous results in vitro and to mimic realistic human NP ingestion exposures. The in vivo model was chosen to model the potential effects of NP consumption at real human intake concentrations on intestinal health, function, and microbial populations. Serial dilutions (1:10) in sterile 18 MΩ DI H_2_O of TiO_2_, SiO_2_, ZnO, and Fe_2_O_3_ NP were prepared to reach 1.4 × 10^−6^ mg/mL, 2.0 × 10^−5^ mg/mL, 9.7 × 10^−6^ mg/mL, and 3.8 × 10^−4^ mg/mL NP, respectively. The osmolality of all solutions was measured using a VAPRO Vapor Pressure Osmometer (Wescor, Logan, UT, USA) to ensure the osmolality < 320 mOsm and to prevent chicken embryo dehydration upon solution injection. Treatment doses of food-grade NP were 1.4 × 10^−6^ mg TiO_2_ NP/mL, 2.0 × 10^−5^ mg SiO_2_ NP/mL, 9.7 × 10^−6^ mg ZnO NP/mL, and 3.8 × 10^−4^ mg Fe_2_O_3_ NP/mL NP, which were extrapolated from realistic human NP intake [7], as summarized in Table 1.

In vitro, physiologically relevant doses of all four NP were formulated considering the daily human intake of NP, NP density, and the previously measured size of food-grade NP [7]. Briefly, the daily intake of TiO_2_ NP was estimated to be 10^11^–10^13^ particles per meal [7]. Considering that the total small intestinal surface area is approximately 2 × 10^6^ cm^2^, ingesting 10^13^ particles exposes the small intestine to 10^6^ particles/cm^2^ in vivo [48]. In the case of SiO_2_ NP, it was found that adults may ingest around 35 mg of fine (0.1–1 µm) or ultrafine (<100 nm) silicate per day, meaning that the human small intestinal tract would be exposed to approximately 10^8^ particles/cm^2^ [34]. For ZnO NP, our group investigated the content of Zn released from cans into the food matrix (10 mg), corresponding to an in vivo dose of 10^7^ particles/cm^2^ [14]. Finally, the concentration of Fe_2_O_3_ NP was extrapolated from the iron content (0.1% *v*/*w*) in a regular portion of meat (200 g), which would be a 10^4^ particles/cm^2^ dose to the small intestine [49]. Table 1 shows order of magnitude-matched doses for this *in ovo* experiment and previous in vitro experiments [7]. Chick small intestinal surface area on embryonic day 17 was estimated to be 10 cm^2^ [46].

### 2.3. Food-Grade Nanoparticles Characterization

Transmission electron microscopy (TEM) was used to measure the primary particle diameter and morphology of the four food-grade NP (TiO_2_, SiO_2_, ZnO, and Fe_2_O_3_). A 10 μL NP suspension (0.1 mg/mL NP) in 18 MΩ sterile DI H_2_O was dispensed on the top of a 400-mesh copper TEM grid (Ted Pella Inc., Redding, CA, USA) and allowed to dry. TEM images of random fields of view were taken using a JEOL JEM-2100F (JEOL, Peabody, MA, USA) and processed with Image J software to measure the diameter of 100 NP. The hydrodynamic size of all four NP was measured using a Nanosight (Malvern Panalytical Ltd., Malvern, UK) and Nanoparticle Tracking Analysis software (NTA). NTA utilizes both light scattering and Brownian motion properties to obtain the NP size distribution of samples in liquid suspension. The zeta potential was evaluated by laser Doppler electrophoresis (LDE) using a Zetasizer Nano ZS90 (Malvern Panalytical Ltd., Malvern, UK). Sample (0.1 mg/mL) measurements were performed in disposable polycarbonate folded capillary cells with gold-plated beryllium–copper electrodes (DTS1070), which were rinsed with 18 MΩ sterile DI H_2_O to remove dust contamination before sample filling. The refractive index used for TiO_2_, SiO_2_, ZnO, and Fe_2_O_3_ NP were the following: 2.42, 1.46, 1.95, and 3.32, respectively. The samples were equilibrated in the instrument chamber for 120 s and measured at 25 °C. Three independent experiments (*n* = 3) were analyzed.

### 2.4. Animals, Intraamniotic Administration, and Tissue Collection

Fertile Cornish cross broiler chicken eggs (*n* = 60) were acquired (Moyer’s Chicks, Quakertown, PA, USA) and incubated at optimal conditions [50] at the Cornell University Animal Science poultry farm incubator. The Cornell University Institutional Animal Care and Use Committee approved all animal protocols (IACUC #2020-0077, approval date: 20 September 2020). The intraamniotic administration procedure was performed as previously described [40,51,52,53]. The amniotic fluid, which consists mainly of water, short peptides, and minerals [54,55], is naturally and orally consumed by the embryo starting at day 17 and is entirely consumed by hatch, which allows for testing the effects of the solution administered into the amniotic fluid, which is wholly consumed shortly after injection [38], on the different systems of interest [40,53,56,57].

On day 17 of embryonic development, eggs with viable embryos were weighed and randomly allocated into six groups (*n* = 10) with approximately equivalent weight distribution. The six treatment groups are as follows: No Injection, 18 MΩ DI H_2_O, 1.4 × 10^−6^ mg TiO_2_ NP/mL, 2.0 × 10^−5^ mg SiO_2_ NP/mL, 9.7 × 10^−6^ mg ZnO NP/mL, and 3.8 × 10^−4^ mg Fe_2_O_3_ NP/mL. 1 mL of solution (per egg) was injected into the amniotic fluid using a sterile 21-gauge needle. 70% ethanol was utilized to sterilize the injection site pre- and post-injection, and cellophane tape was used to seal the site. The eggs were then placed in hatching baskets, with each treatment group equally represented at each location within the same incubator. Upon hatch (Day 21), as previously described [40,42,58], the birds were weighed and euthanized by CO_2_ exposure. Blood was collected in heparinized tubes (ThermoFisher Scientific, Waltham, MA, USA). The small intestine, cecum, pectoral muscle, and liver were obtained and stored in separate sterile cryovials (Simport, Beloeil, Canada). Tissue samples were frozen immediately in liquid nitrogen and stored at −80 °C until analysis.

### 2.5. Blood Hemoglobin Quantification

Blood hemoglobin concentrations were determined using the QuantiChrom^TM^ Hemoglobin Assay (BioAssay Systems, Hayward, CA, USA) per the manufacturer’s instructions.

### 2.6. Glycogen Concentration Analysis as Measurement of Energetic Status

The pectoralis muscle and liver glycogen content analysis was performed as previously described [53,59,60,61]. Detailed methodology is described in the Supplementary Materials.

### 2.7. Total RNA Isolation and Real-Time Polymerase Chain Reaction (RT-qPCR)

These procedures were conducted as was previously described [40,42,53,59], and the primers utilized are below in Table 2. Detailed methodology is described in the Supplementary Materials.

### 2.8. Microbial Sample Collection, Cecal Content DNA Isolation, Polymerase Chain Reaction Amplification of 16s rDNA

These procedures were conducted as previously described [40,42,62]. Detailed methodology is provided in the Supplementary Materials.

### 2.9. Duodenal Tissue Morphology Examination

**Figure 1:**
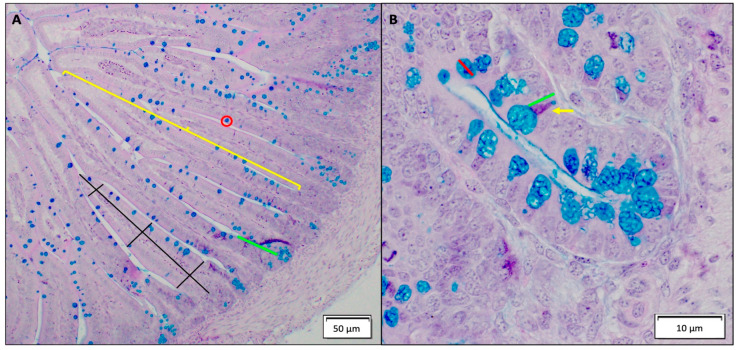
(**A**) Histomorphological depiction of a normal small intestine. Enterocytes are located within the small intestine villi, and goblet cells are the blue cells within the enterocyte (red circle). The crypt villus axis is represented with a yellow bar demonstrating where the axis is located. The following variables were measured: villus surface area (depicted in black) and crypt depth (depicted in green). (**B**) Histomorphological depiction of a normal small intestinal crypt. Paneth cells (yellow arrow, pyramidal shape) play a key role in innate intestinal immunity and homeostasis. The following variables were measured: goblet cell diameter (red) and Paneth cell diameter (green). The type of goblet cell shown for the goblet cell diameter measurement example represents an acidic goblet cell: the predominant goblet cell type found in the study.

Intestinal morphology analysis was performed on duodenal sections as previously described [53,56,59]. Sections were fixed in fresh 4% (*v*/*v*) buffered formaldehyde, dehydrated, cleared. and embedded in paraffin. Sections were cut serially (5 μm thickness) and put onto glass slides. Sections were deparaffinized in xylene, rehydrated in graded alcohol series, and stained with Alcian Blue/Periodic acid-Schiff. The following variables (shown in Figure 1) were measured via light microscopy (EPIX XCAP software, Olympus, Waltham, MA, USA): villus height and width, crypt depth, goblet cell diameter, goblet cell type and count within the villus and crypt, Paneth cell number per crypt, and Paneth cell width. Five biological samples per treatment group (*n* = 5) and four segments per biological sample were analyzed. Ten randomly selected villi and crypts were analyzed per segment. Cell size measurements and counts were counted in ten randomly selected villi and/or crypts per segment (40 replicates per biological sample). The villus surface area was calculated as previously described [63].

### 2.10. Statistical Analysis

Results are presented as mean ± standard error of the mean (*n* ≥ 5) in tables and heatmaps. Microsoft Excel (Microsoft Corporation, Redmond, WA, USA) was utilized to create graphs and heatmaps (based on conditional formatting using color scales). The Shapiro–Wilk test was utilized to assess distribution normality. Results were analyzed by one-way ANOVA with a post hoc Duncan test to compare differences between treatment groups, with results considered statistically significant at *p* < 0.05. Statistical analyses were performed with R version 4.0.4 software.

## 3. Results

### 3.1. Food-Grade Metal Oxide Nanoparticle Characterization

**Figure 2:**
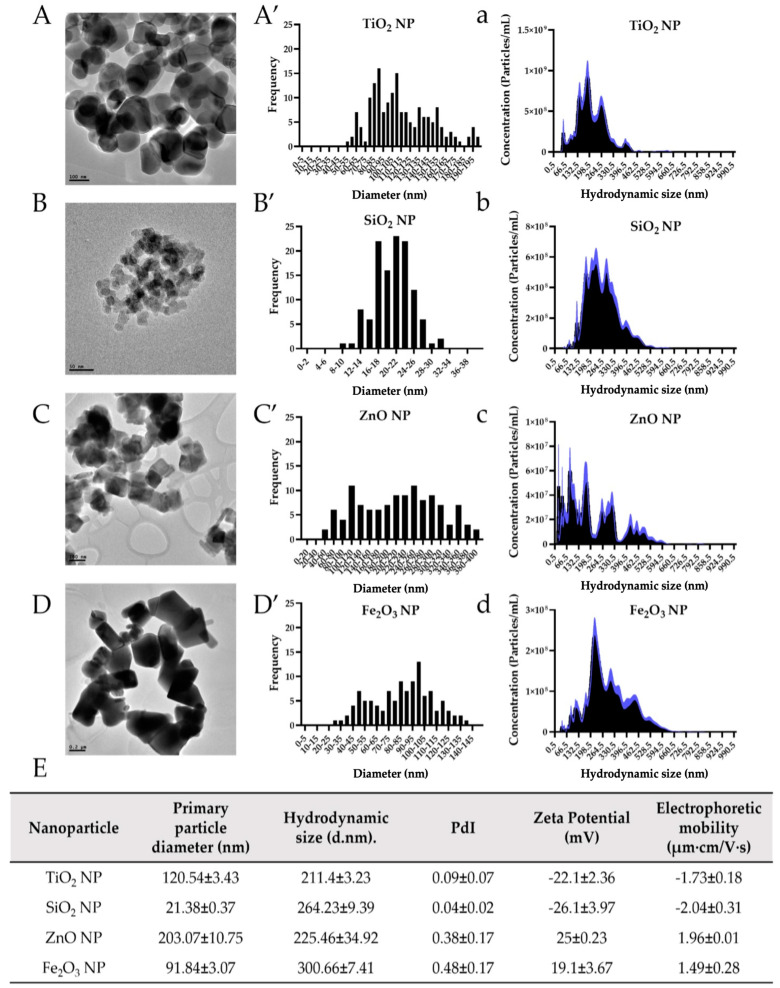
Food-grade nanoparticles (NP) characterization. Transmission electron microscopy (TEM) images of TiO_2_ NP (**A**), SiO_2_ NP (**B**), ZnO NP (**C**), and Fe_2_O_3_ NP (**D**) in dry form. Frequency histogram of the primary particle diameter (nm) measured from TEM images (**A’**−**D’**). Particle size distribution of hydrated NP measured by NanoSight nanoparticle tracking analysis (NTA) technique using dynamic light scattering (**a**–**d**). Table summarizing the average of the primary particle diameter (nm), hydrodynamic size (nm), polydispersity index (PdI), zeta potential values (mV), and electrophoretic mobility (µm·cm/V·s) of each NP (**E**). Data are shown as mean ± SEM.

TEM, NTA, DLS, and LDE techniques were utilized to fully characterize all four food-grade NP to understand NP behavior and biotransformation in our in ovo system. TEM images were taken of NP suspension at 0.1 mg/mL, which shows each food-grade NP’s shape in its dry form (Figure 2A–D). Agglomeration events occur in all four food-grade NP preparations as primary NP can be seen interacting and attaching in clusters. Using an image processing program (ImageJ), the primary particle size of food-grade TiO_2_, SiO_2_, ZnO, and Fe_2_O_3_ NP were approximately measured to be 120, 21, 203, and 92 nm, respectively (Figure 2E). NTA was used to measure NP size in its hydrated form (hydrodynamic size).

As expected, all four food-grade NP slightly agglomerated when suspended in sterile 18 MΩ nanopure H_2_O, averaging a hydrodynamic size of 211 nm (TiO_2_ NP), 264 nm (SiO_2_ NP), 225 nm (ZnO NP) and 301 nm (Fe_2_O_3_ NP). The polydispersity index (PdI) was also calculated. This is a dimensionless measure of the broadness of the size distribution calculated from the cumulants analysis, where values ranged from 0 to 1, with <0.05 being very monodisperse, <0.08 nearly monodisperse, 0.08 to 0.7 mid-range polydisperse, and >0.7 very polydisperse. Thus, our results indicate that both TiO_2_ (PdI = 0.09) and SiO_2_ NP (PdI = 0.04) are more monodispersed than ZnO (PdI = 0.38) and Fe_2_O_3_ NP (PdI = 0.48). This tendency is clearly depicted in the particle size distribution graphs of hydrated NP (Figure 2a–d).

Finally, the NP zeta potential (mV) and the electrophoretic mobility (µm·cm/V·s) were measured using the laser Doppler electrophoresis technique (LDE), where the stability and mobility of a particle suspended in a liquid solution were measured under an applied electric field. As the higher the zeta potential value, the more stable the NP dispersion, and considering that ±30 mV is the best stability value for aqueous systems, all four food-grade NP presented mid-range to good stability values (Figure 2E). Moreover, while both TiO_2_ (−22 mV) and SiO_2_ NP (−26 mV) were found to be negatively charged, both ZnO (25 mV) and Fe_2_O_3_ NP (19 mV) presented a positive surface charge. The electrophoretic mobility of food-grade TiO_2_, SiO_2_, ZnO, and Fe_2_O_3_ NP was −1.73, −2.04, 1.96, and 1.49 µm·cm/V·s, respectively.

### 3.2. Gross Physiological Parameters

No significant differences in body weight, cecum weight, or cecum-to-body weight ratios were found between treatment groups (Table 3).

### 3.3. Hemoglobin and Glycogen Concentrations

The average hemoglobin concentration was significantly decreased in the food-grade NP-treated and H_2_O-injected groups when compared with the NI group (*p* < 0.05, Table 4). Pectoral muscle glycogen content was increased in the TiO_2_ NP and SiO_2_ NP treated groups compared to the controls (*p* < 0.05). For liver glycogen content, there were no significant differences between the treatment groups.

### 3.4. Duodenal Gene Expression of Fe, Zn, Brush Border Membrane Functionality, and Inflammation Related Proteins

#### 3.4.1. Fe- and Zn-Related Proteins

**Figure 3:**
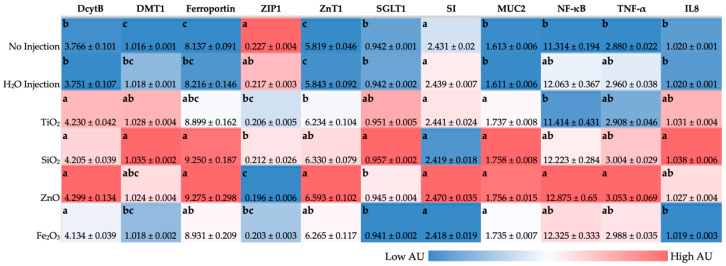
Effect of the intraamniotic administration of food-grade nanoparticles (NP) on duodenal gene expression of nutrient transporters, brush border membrane functional proteins, and pro-inflammatory cytokines. Gene expression has been normalized to the 18S housekeeping gene. Values are the means ± SEM, *n* = 6. Per gene (in the same column), blue depicts lower gene expression levels, and red depicts higher gene expression levels. ^a–c^ Per gene (in the same column), treatment groups not indicated by the same letter are significantly different according to one-way ANOVA with post hoc Duncan test (*p* < 0.05). NI = non-injected. DcytB, Duodenal cytochrome b; DMT1, Divalent metal transporter 1; ZIP1, Zinc Transport Protein 1; ZnT1, Zinc transporter 1; SGLT1, Sodium-glucose cotransporter 1; SI, Sucrase isomaltase; MUC2, Mucin 2; NF-_Κ_B, Nuclear factor Kappa B Subunit 1; TNF-α, Tumor necrosis factor-alpha; IL8, Interleukin 8.

For the proteins responsible for Fe uptake (Figure 3), duodenal cytochrome b (DcytB) gene expression was significantly upregulated (*p* < 0.05) in all food-grade NP-exposed groups when compared to the controls. Divalent metal transporter 1 (DMT1) expression was upregulated with TiO_2_ and SiO_2_ NP exposure when compared to the NI control. ZnO and Fe_2_O_3_ NP exposure did not result in significant DMT1 expression changes when compared with the NI and H_2_O controls. When compared to the NI control, ferroportin expression was significantly upregulated with SiO_2_, ZnO, and Fe_2_O_3_ NP exposure. For proteins related to cellular Zn uptake (Figure 3), transport, and storage, the gene expression of Zn Transport Protein 1 (ZIP1) was significantly downregulated (*p* < 0.05) with food-grade NP exposure when compared to the NI control. Food-grade NP exposure resulted in significant upregulation of Zn transporter 1 (ZnT1) gene expression (*p* < 0.05) compared with the controls.

#### 3.4.2. BBM Functionality, Mucin Production, and Pro-Inflammatory Proteins

Sodium-glucose cotransporter 1 (SGLT1) expression was upregulated (Figure 3, *p* < 0.05) in the presence of SiO_2_ NP when compared with the controls. Mucin 2 (MUC2) expression was significantly upregulated (*p* < 0.05) with NP exposure when compared with the controls. Interleukin-8 (IL8) expression was upregulated (*p* < 0.05) in the presence of SiO_2_ NP when compared with the controls. No significant differences in gene expression of sucrase-isomaltase (SI), nuclear transcription factor (NF-κβ), and tumor necrosis factor (TNF-α) were found when comparing the food-grade NP exposed groups to the controls.

### 3.5. Intestinal Content Bacterial Genera- and Species-Level Analysis

**Figure 4:**
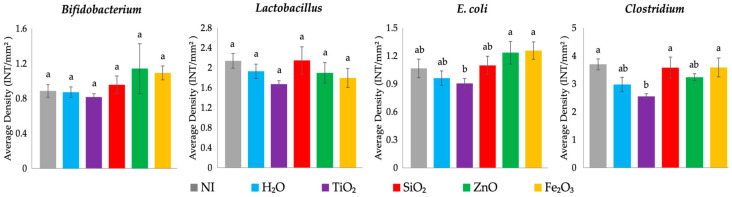
Effects of intraamniotic injections of food-grade NP on cecal genera (*Bifidobacterium*, *Lactobacillus*, and *Clostridium*) and species-level bacterial populations (*E. coli*) on day of hatch. Values are presented as means ± SEM, *n* = 5. ^a,b^ Per bacterial category, treatment groups that do not share letters significantly differ according to one-way ANOVA with post hoc Duncan test (*p* < 0.05). NI = non-injected.

TiO_2_ NP exposure resulted in a significantly decreased relative abundance of *Clostridium* spp., an opportunistic or potentially pathogenic bacteria, compared with the NI control, SiO_2_, and Fe_2_O_3_ treated groups (*p* < 0.05, Figure 4). *E. coli*, a possibly pathogenic bacteria, was significantly decreased with TiO_2_ exposure compared with the ZnO and Fe_2_O_3_ exposed groups (*p* < 0.05, Figure 4). There were no significant differences between *Bifidobacterium* and *Lactobacillus* spp. relative abundance between the treatment groups.

### 3.6. Morphometric Analysis of Duodenal Villi, Depth of Crypts, Goblet Cells, and Paneth Cells

TiO_2_ and ZnO NP exposure resulted in a significantly decreased (*p* < 0.05, Table 5) villus surface area when compared with all other treatment groups. In contrast, Fe_2_O_3_ and SiO_2_ NP exposure did not result in significant differences in villus surface area compared with the NI and H_2_O controls. The crypt depth of the ZnO and Fe_2_O_3_ NP exposed groups was significantly decreased (*p* < 0.05) when compared with all other treatment groups. TiO_2_ and SiO_2_ NP exposure resulted in a significantly decreased (*p* < 0.05) crypt depth when compared with the NI and H_2_O controls and significantly increased (*p* < 0.05) crypt depth when compared with ZnO and Fe_2_O_3_ NP exposed groups.

Food-grade NP treatment resulted in a significantly smaller villi goblet cell diameter (*p* < 0.05) when compared to the H_2_O control (Table 6). The acidic villi goblet cell number was significantly lower (*p* < 0.05) in the TiO_2_, SiO_2_, and ZnO NP exposed groups when compared with the NI, H_2_O, and Fe_2_O_3_ NP groups. There were no significant differences in neutral villi goblet cell number between groups. The Fe_2_O_3_ NP treatment group had the highest mixture villi goblet cell number when compared with all other groups, and TiO_2_ and ZnO NP exposed groups had a significantly higher (*p* < 0.05) mixture villi goblet cell number when compared with the H_2_O control. The total villi goblet cell number was significantly lower (*p* < 0.05) in the TiO_2_, SiO_2_, and ZnO NP exposed groups when compared with the NI, H_2_O, and Fe_2_O_3_ NP groups.

The crypt goblet cell diameter of the food-grade NP treatment groups was significantly lower (*p* < 0.05, Table 7) when compared with the H_2_O control. The Fe_2_O_3_ NP exposed group had a significantly higher (*p* < 0.05) total crypt goblet cell number and acidic crypt goblet cell number when compared to the H_2_O control. When comparing the total crypt goblet cell number and acidic crypt goblet cell number in the TiO_2_, SiO_2_, and ZnO NP exposed groups to the NI and H_2_O controls, no significant differences were found (Table 5). Between groups, no significant differences in the neutral crypt goblet cell number were found. NP exposure resulted in a significantly decreased (*p* < 0.05) mixture of crypt goblet cell numbers when compared with the controls.

The average number of Paneth cells per crypt was significantly increased (*p* < 0.05) in all food-grade NP-treated groups when compared to the controls, where TiO_2_ NP exposure resulted in the highest average number of Paneth cells per crypt versus exposure relative to all other tested NP types (Table 8). Crypt Paneth cell diameter was significantly increased (*p* < 0.05) in the TiO_2_, SiO_2_, and ZnO NP exposed groups when compared to the NI and H_2_O controls. With Fe_2_O_3_ NP exposure, crypt Paneth cell diameter was not significantly increased when compared with the NI control.

## 4. Discussion

NP are widely used as food additives within the food industry, where TiO_2_ and SiO_2_ NP are commonly used as food coloring or anti-caking agents, and ZnO and Fe₃O₂ NP are added as antimicrobials and coloring agents, respectively, and can be used as micronutrient supplements. Food additive NP exposure effects on GI functionality and development are still unclear; thus, our study aimed to assess the potential effects of physiologically relevant dosages of food-grade NP ingestion in vivo. Our well-established in vivo model of *G. gallus* was previously utilized to elucidate the effects of ingesting functional foods and food additives at physiologically relevant doses [38,39,41,42]. In this in vivo model, chemical-grade TiO_2_ and SiO_2_ NP were previously demonstrated to have the potential to negatively affect intestinal functionality and health [40]. Due to the widespread consumption of foods containing NP, our present study builds upon our previous study of chemical-grade NP to assess the effects of commonly utilized food-grade metal oxide NP. This present study utilized the intraamniotic administration approach in the *G. gallus* model to assess the effect of food-grade metal oxide NP (TiO_2_, SiO_2_, ZnO, and Fe_2_O_3_) at human-relevant doses on duodenal BBM development and functionality (gene expression and histomorphology) and the relative abundance of representative cecal microbial species immediately post-hatch. These results show that food-grade metal oxide NP delivered via intraamniotic administration affected BBM functional gene expression, intestinal development, and cecal bacterial populations.

NP are well-known to be more reactive with the environment than their bulk and chemical-grade counterparts, mainly because of their size-specific physicochemical properties [2]. Previous studies have described how different environments with different chemical properties can provoke substantial changes in NP suspension, such as erosion, ion release, agglomeration or aggregation phenomenon, protein corona formation, and attractive and repulsive forces [64,65]. In our previous studies, we found that more complex solutions with high protein content, such as DMEM (Dulbecco’s Modified Eagle Medium) cell culture media, fetal bovine serum (FBS), and in vitro gastric digesta as well as changes in the pH, and food matrices have a direct influence in NP physicochemical changes such as agglomeration and dissolution (ionic release) [7,35,36]. These changes can drastically impact NP interaction with live organisms, with NP eventually becoming more hazardous. For example, while agglomerations of TiO_2_ NP suspended in cell culture media (DMEM + FBS) caused structural damage to the in vitro epithelium, the release of ions from ZnO NP in cell culture medium impaired gut-derived bacteria (e.g., *E. coli* and *L. rhamnosus*) and altered the activity of in vitro digestive brush border enzymes (e.g., intestinal alkaline phosphatase, aminopeptidase-N, and sucrase-isomaltase) [7,31]. An extensive investigation into the effects of in vitro digestion on several engineered NP found that while TiO_2_ (0.42%) and Fe_2_O_3_ NP (2.27%) showed minor dissolution in simulated cascade digestion (saliva to gastric to intestinal fluids), SiO_2_ and ZnO NP were partially (65.5%) and fully (100%) dissolved because of the drastic pH reduction in the stomach phase, respectively, although Si^4+^ ions, but not ZnO, were found to reprecipitate in the intestinal phase [66]. Therefore, from a toxicological point of view, NP biotransformation is an essential factor in further investigating and unveiling any potential effects from exposure. Zn and Fe ionic controls did not result in human cell cytotoxicity, statistically significant decreases in bacterial viability, or changes in brush border membrane (BBM) enzyme activity in previous in vitro work [7]; therefore, we did not include these conditions in the current study. As many studies investigating NP environmental or biological interactions typically utilize ultrasonication procedures to prepare test suspensions from commercially available NP powders, we have followed both OECD and NIST guidelines as well as standardized protocols for NP sonication (NanoGenotox) to facilitate reproducibility among studies [47,67,68]. Therefore, after the sonication procedure whereby the power delivered to the sample was 7.32 W, all four food-grade NP were fully characterized by TEM, DLS, NTA, and LDE. While the primary particle size of TiO_2_, SiO_2_, ZnO, and Fe_2_O_3_ NP was 120, 21, 203, and 92 nm, respectively, the values slightly increased when the NP size was measured in the nanopure H_2_O suspension by NTA. Most metal oxide NP (e.g., TiO_2_, ZnO, CeO, and Fe_2_O_3_ NP) form relatively large clusters or agglomerates once they are hydrated in nanopure H_2_O and/or natural aqueous matrices [64,69]. This agglomeration is consistent with the principles of colloidal chemistry and is expected to be strongly influenced by the ionic strength and pH of aqueous environments in which NP are suspended [70]. By DLS, French et al. (2009) showed that TiO_2_ NP between 4 to 5 nm readily formed stable aggregates between 20 and 1000 nm in an aqueous suspension adjusted to a high ionic strength of 0.0165 M [70]. In this study, food additive TiO_2_, SiO_2_, ZnO, and Fe_2_O_3_ NP were suspended and sonicated in sterile nanopure H_2_O, which presented low ionic strength and low buffering capacity, and its hydrodynamic size also increased up to 211, 264, 225, and 301 nm, respectively. This could be explained by particles colliding and heating during sonication, which can influence NP properties such as morphology, surface oxide characteristics, and surface charge. Moreover, many commercially available NP powders were synthesized using high-temperature vapor phase processes, producing droplets that may coalesce fully or partially with others and form larger primary particles that are often too strong to break via sonication [46].

In previous intraamniotic administration studies, the downregulation of Fe duodenal transporter genes was associated with improved Fe status, where due to Fe-sufficient conditions, additional Fe transporters were not necessary as a mechanism to compensate for Fe insufficiency [42,53]. In line with our previous in vivo and in vitro studies, the administration of tested food-grade metal oxide NP generally upregulated DcytB, DMT1, and ferroportin, suggesting Fe insufficient luminal and basolateral conditions [37,40,56]. For the expression of proteins related to cellular Zn uptake, transport, and storage, the gene expression of ZIP1 was significantly downregulated with food additive NP exposure compared with the NI control (Figure 3). ZIP1 upregulation has been associated with Zn abundant and deficient conditions—ZIP1 is present in cellular organelles in Zn abundant media, but only on the cell membrane surface when Zn is deficient [71,72]. ZnT1 is located on the basolateral side of enterocytes and exports Zn^2+^ from inside the enterocyte into circulation [72,73,74]. Food-grade NP exposure resulted in significantly upregulated ZnT1 gene expression compared with the controls, in agreement with our previous in vivo and in vitro studies where ZnT1 upregulation was associated with NP administration [14,40]. ZnO NP solubilizes in acidic conditions, such as in the intestinal lumen, and can provide bioavailable Zn to the embryo, resulting in the upregulation of ZnT1 and downregulation of ZIP1 [14,71,75]. Additionally, food additive ZnO NP exposure resulted in the significant upregulation of pro-inflammatory genes TNF-α and NF-κB (Figure 3). Nuclear Factor kappa beta (NF-κB) is a transcriptional factor activated by various intra- and extra-cellular stimuli such as cytokines, reactive oxygen species (ROS), or bacterial metabolites. When NF-κB is stimulated, it translocates within the cell nucleus and is involved in various biological functions, including the release of proinflammatory mediators, such as tumor necrosis factor-alpha (TNF-α) and interleukins [76,77]. TNF-α is an immune cell regulator, while interleukins are a group of cytokines that play a physiological role in inflammation and a pathological role in systemic inflammatory states. On one side, changes in Zn metabolism gene expression are associated with pro-inflammatory conditions, which have previously been shown to increase Zn absorption, and on the other hand, a major target of Zn is NF-κB, where Zn is capable of modulating NF-κB activity, and NF-κB expression positively correlates with TNF-α expression [40,56,78,79,80]. Alterations in mineral transporter expression can potentially be attributed to food-grade NP agglomeration, where NP agglomerates were observed to settle down in the BBM in an in vitro model of the GI tract, resulting in significantly fewer microvilli in the NP treatment conditions, indicative of reduced BBM development [7]. SGLT1 gene expression was upregulated with food additive TiO_2_ and SiO_2_ NP exposure (Figure 3). In previous studies, an upregulation in BBM functional gene expression suggested an improvement in *G. gallus* hatchling intestinal development, digestive capabilities, and the potential for increased micronutrient (Fe or Zn) absorption [81]; however, a previous in vivo study on chemical-grade NP exposure resulted in increased expression of BBM functional genes, which the authors posited may be indicative of intestinal development or of a compensatory mechanism to improve absorption due to intestinal damage [40].

The intraamniotic administration of food-grade metal oxide NP impacted intestinal development. Exposure to food additive TiO_2_, SiO_2_, and ZnO NP resulted in a decrease in villus surface area (Table 5), potentially indicative of decreased digestive enzyme and absorptive capacity [82]. Crypt depth was found to be significantly shallower for all food additives NP-exposed groups when compared to the controls (Table 5), where NP exposure was shown to previously be associated with increased enterocyte proliferation, either resulting from a compensatory mechanism for intestinal damage with TiO_2_ and SiO_2_ NP exposure, or nutritionally beneficial ZnO and Fe_2_O_3_ NP exposure [83,84,85]. Similarly, Khajeh Bami et al. (2018) found that 42 days of ZnO NP exposure resulted in decreased crypt depth in *G. gallus* [85]. This could indicate that the broilers were utilizing food-grade ZnO and Fe_2_O_3_ NP for nutritional benefit, which could be supported by IL8 downregulation compared to SiO_2_ and TiO_2_ NP exposure (Figure 3), which indicates a decreased level of systemic inflammation due to IL-8 mediation of the inflammatory immune response.

The major goblet cell mucins in the small and large intestines are MUC2 proteins, which are gel-forming secretory mucins that bind with intestinal bacteria and are associated with facilitating hydrolysis and absorption of nutrients [36,57,86,87]. Paneth cells secrete antimicrobial peptides into mucin produced by goblet cells, and MUC2 gene expression correlates with increased Paneth cell number and size [88]. MUC2 gene expression was significantly upregulated with NP exposure, though a significant reduction in villi goblet cell diameter was associated with NP treatment compared with the H_2_O injection control (Figure 3). Taken together, this could be due to NP exposure-induced alterations in gut microbiota populations, resulting in NP biofilm formation on the intestinal mucosa surface, potentially hindering the ability of goblet cells to produce mucin, and where MUC2 upregulation could be a compensatory mechanism to prevent intestinal damage [36]. A decrease in total villi goblet cells, specifically acidic villi goblet cells, was observed with food-grade TiO_2_, SiO_2,_ and Zn NP exposure (Table 6), indicating a decrease in synthesis and secretion of acidic luminal mucin by duodenal goblet cells [38,89]. With food additive Fe_2_O_3_ NP exposure, a significant increase in the total villi goblet cell number and a significant increase in the proportion of acidic and increase in mixture goblet cells was found in comparison with other food additive NP treated groups, with total villi acidic goblet cells maintained versus the controls (Table 6). In addition to serving as a protective intestinal epithelial barrier, this mucin also functions as a habitat that supports probiotic bacterial proliferation, increased bacterial metabolite production, and the promotion of epithelial cell function [86,90].

Previous in vitro and in vivo studies have shown that metal oxide exposure can lead to compositional shifts in GI bacterial populations abundance [91]. ZnO and Fe_2_O_3_ NP are utilized for mineral fortification of foods, and exposure to these potentially nutritional NP resulted in an increased population of health-promoting bacteria, *Bifidobacterium*, though not statistically significant. Further, food additive ZnO and Fe_2_O_3_ NP exposure was associated with a significant upregulation of MUC2 gene expression and increased Paneth cell number per crypt, which can be associated with providing an environment conducive to *Bifidobacterium* spp. proliferation, attributed to increased acidic mucin production [92,93]. The potentially pathogenic *Clostridium* spp. and *E. coli* were increased following exposure to food-grade SiO_2_, ZnO, or Fe_2_O_3_ NP compared with the H_2_O control (Figure 4). Gene expression resulting from food additive SiO_2_, ZnO, or Fe_2_O_3_ NP exposure demonstrated an increase in pro-inflammatory cytokine expression, NF-κB and TNF-α (Figure 3), and a significantly increased number of Paneth cells per crypt (Table 8), which can be linked with the increased abundance of potentially opportunistic *Clostridium* spp. and possibly pathogenic *E. coli* (Figure 4). Previous work has shown that SiO_2_ and ZnO NP possessed antimicrobial properties; however, in this current study, food-grade NP were used where the dosage was too low or NP was too large to be antimicrobial [7,34,40,94]. Food additive TiO_2_ NP exposure was associated with decreased populations of Gram-positive (*Bifidobacterium* spp., *Lacticaseibacillus* spp., and *Clostridium* spp.) and Gram-negative (*E. coli*) bacteria (Figure 4), where TiO_2_ NP exposure has previously been found to lead to gut microbiota dysbiosis and decreased abundance of beneficial *Bifidobacterium* and *Lacticaseibacillus* genera [95,96]. Additional studies are warranted to assess cecal microbiota shifts, intestinal functionality, and developmental changes post-hatch and during a long-term feeding trial with human-relevant dosages of food-grade NP. The potential hazards and benefits of the tested food additive NP should be further explored to determine a relevant dose at which the potential associated risks and benefits are balanced. Further, future studies should clarify if results associated with NP exposure could be associated with the NP particles themselves or NP ions alone.

## 5. Conclusions

This study demonstrates that the type of food-grade metal oxide ingested can affect intestinal development and functionality, and cecal bacteria content and abundance, in vivo (*G. gallus*). Our results suggest that food additive TiO_2_ and SiO_2_ NP have the potential to negatively affect intestinal functionality. Food additive ZnO NP administration effects were associated with supporting intestinal development or compensatory mechanisms due to intestinal damage. Further, food additive Fe_2_O_3_ NP were found to be a potential option for Fe fortification, though with potential perturbations in intestinal functionality and development. Our NP intraamniotic administration results are generally concurrent with previous in vivo and in vitro studies with the same types and concentrations of chemical- and food-grade NP, which validates the use of these methods as a relatively fast and low-cost screening method for elucidating the potential perturbations of NP ingestion on microbiota composition and intestinal functionality and development. However, the lack of microscale particle controls is a limitation of the study. Given that the current study establishes a baseline of effects associated with physiologically relevant concentrations of food-grade metal oxide NP ingestion and that human exposure to NP through food, water, and the environment is inevitable, consequences of food-grade metal oxide NP ingestion, such as the penetration of NP across cell and tissue barriers, should be further evaluated in long-term animal feeding trials.

## Figures and Tables

**Table 1:** Physiologically relevant nanoparticle (NP) doses extrapolated from the real human intake used in vitro studies and the current *in ovo* study expressed as mg/mL and NP/cm^2^.

Nanoparticles (NP) Doses								
	TiO_2_ NP		SiO_2_ NP		ZnO NP		Fe_2_O_3_ NP	
	Dose	NP/cm^2^	Dose	NP/cm^2^	Dose	NP/cm^2^	Dose	NP/cm^2^
In vivo dose	10^13^ NP/day [35]	5 × 10^−6^	35 mg/day [34]	1 × 10^−8^	10 mg/day [14]	6 × 10^−7^	200 mg/day [7]	4 × 10^−4^
In vitro dose ^1^	1.4 × 10^−6^ mg/mL	7 × 10^−6^	2 × 10^−5^ mg/mL	5 × 10^−8^	9.7 × 10^−6^ mg/mL	4 × 10^−7^	3.8 × 10^−4^ mg/mL	4 × 10^−4^
*In ovo* dose	1.4 × 10^−6^ mg/mL	2 × 10^−6^	2 × 10^−5^ mg/mL	2 × 10^−8^	9.7 × 10^−6^ mg/mL	1 × 10^−7^	3.8 × 10^−4^ mg/mL	1 × 10^−4^

^1^ In vitro doses from García-Rodríguez et al. [7]

**Table 2:** Real-time polymerase chain reaction (RT-PCR) primer sequences.

Gene	Forward Primer (5′-3′)	Reverse Primer (5′-3′)	Base Pairs	GI Identifier
DcytB	CATGTGCATTCTCTTCCAAAGTC	CTCCTTGGTGACCGCATTAT	103	20380692
DMT1	TTGATTCAGAGCCTCCCATTAG	GCGAGGAGTAGGCTTGTATTT	101	20659748
Ferroportin	CTCAGCAATCACTGGCATCA	ACTGGGCAACTCCAGAAATAAG	98	61098365
ZIP1	TGCCTCAGTTTCCCTCAC	GGCTCTTAAGGGCACTTCT	144	107055139
ZnT1	GGTAACAGAGCTGCCTTAACT	GGTAACAGAGCTGCCTTAACT	105	54109718
SGLT1	GCATCCTTACTCTGTGGTACTG	TATCCGCACATCACACATCC	106	8346783
SI	CCAGCAATGCCAGCATATTG	CGGTTTCTCCTTACCACTTCTT	95	2246388
MUC2	ATTGTGGTAACACCAACATTCATC	CTTTATAATGTCAGCACCAACTTCTC	134	423101
NF-κB	CACAGCTGGAGGGAAGTAAAT	TTGAGTAAGGAAGTGAGGTTGAG	100	2130627
TNF-α	GACAGCCTATGCCAACAAGTA	TTACAGGAAGGGCAACTCATC	109	53854909
IL-8	TCATCCATCCCAAGTTCATTCA	GACACACTTCTCTGCCATCTT	105	395872
18S	GCAAGACGAACTAAAGCGAAAG	TCGGAACTACGACGGTATCT	100	7262899

DcytB, Duodenal cytochrome b; DMT1, Divalent metal transporter 1; ZIP1, Zinc Transport Protein 1; ZnT1, Zinc transporter 1; SGLT1, Sodium-glucose cotransporter 1; SI, Sucrase isomaltase; MUC2, Mucin 2; NF-_Κ_B, Nuclear factor Kappa B Subunit 1; TNF-α, Tumor necrosis factor-alpha; IL8, Interleukin 8; 18S rRNA, 18S Ribosomal subunit.

**Table 3:** Body weight, cecum weight, and cecum:body weight ratio in all groups ^1^.

Treatment Group	Body Weight (g)	Cecum (g)	Cecum: BW
NI	42.06 ± 1.35 ᵃ	0.45 ± 0.05 ᵃ	0.011 ± 0.002 ᵃ
H_2_O	42.16 ± 1.28 ᵃ	0.29 ± 0.04 ᵃ	0.007 ± 0.001 ᵃ
TiO_2_	42.29 ± 0.80 ᵃ	0.39 ± 0.07 ᵃ	0.009 ± 0.002 ᵃ
SiO_2_	42.98 ± 1.34 ᵃ	0.32 ± 0.03 ᵃ	0.008 ± 0.001 ᵃ
ZnO	43.19 ± 0.92 ᵃ	0.32 ± 0.04 ᵃ	0.007 ± 0.001 ᵃ
Fe_2_O_3_	43.12 ± 0.92 ᵃ	0.41 ± 0.05 ᵃ	0.010 ± 0.001 ᵃ

^1^ Values are means ± SEM, *n* = 8–11. ^a^ Treatment groups not indicated by the same letter in the same column are significantly different according to one-way ANOVA with post hoc Duncan test (*p* < 0.05). NI = non-injected.

**Table 4:** Blood hemoglobin concentrations (g/dL) and pectoral muscle glycogen concentrations (mg/g) following genistein exposure ^1^.

Treatment Group	Average Hemoglobin (g/dL)	Average Pectoral Glycogen (mg/g)	Average Liver Glycogen (mg/g)
NI	12.52 ± 0.91 ^a^	0.01 ± 0.00 ^b^	0.08 ± 0.03 ᵃ
H_2_O	10.13 ± 0.71 ^b^	0.02 ± 0.00 ^b^	0.24 ± 0.05 ᵃ
TiO_2_	9.42 ± 1.00 ^b^	0.08 ± 0.02 ^a^	0.05 ± 0.02 ᵃ
SiO_2_	8.07 ± 0.33 ^b^	0.12 ± 0.01 ^a^	0.06 ± 0.04 ᵃ
ZnO	10.03 ± 0.78 ^b^	0.03 ± 0.01 ^b^	0.25 ± 0.06 ᵃ
Fe_2_O_3_	8.04 ± 0.51 ^b^	0.02 ± 0.01 ^b^	0.23 ± 0.06 ᵃ

^1^ Values are means ± SEM, *n* = 6–12. ^a,b^ Treatment groups not indicated by the same letter in the same column are significantly different (*p* < 0.05) according to one-way ANOVA with post hoc Duncan test.

**Table 5:** Effects of intraamniotic administration of experimental food-grade NP solutions on duodenal small intestinal villi and depth of crypts ^1^.

Treatment Group	Villus Surface Area (µm^2^)	Crypt Depth (µm)
NI	168.19 ± 3.72 ᵃᵇ	45.77 ± 1.32 ᵇ
H_2_O	171.45 ± 4.20 ᵃᵇ	50.73 ± 1.10 ᵃ
TiO_2_	135.31 ± 3.60 ᶜ	34.77 ± 0.60 ᶜ
SiO_2_	161.37 ± 4.12 ᵇ	30.21 ± 0.70 ᵈ
ZnO	144.38 ± 3.39 ᶜ	25.47 ± 0.76 ᵉ
Fe_2_O_3_	176.38 ± 6.43 ᵃ	25.87 ± 0.73 ᵉ

^1^ Values are means ± SEM, n = 5. ^a–e^ Treatment groups not indicated by the same letter in the same column are significantly different (*p* < 0.05) according to one-way ANOVA with post hoc Duncan test. NI = non-injected.

**Table 6:** Effects of intraamniotic administration of experimental food-grade NP solutions on villi goblet cell diameter, goblet cell count, and goblet cell type ^1^.

Treatment Group	Villi Goblet Cell Diameter (µm)	Villi Goblet Cell Number (Unit)			
		Acidic	Neutral	Mixture	Total
NI	4.65 ± 0.06 ᵇ	36.76 ± 0.85 ᵃ	0.02 ± 0.01 ᵃ	0.30 ± 0.06 ᵇ	37.08 ± 0.86 ᵃ
H_2_O	5.13 ± 0.06 ᵃ	32.92 ± 0.68 ᵇ	0.00 ± 0.00 ᵃ	0.05 ± 0.03 ᶜ	32.97 ± 0.68 ᵇ
TiO_2_	3.63 ± 0.06 ᶜ	26.12 ± 0.56 ᶜ	0.00 ± 0.00 ᵃ	0.22 ± 0.05 ᵇ	26.34 ± 0.57 ᶜ
SiO_2_	3.67 ± 0.06 ᶜ	26.92 ± 0.52 ᶜ	0.00 ± 0.00 ᵃ	0.06 ± 0.02 ᶜ	26.97 ± 0.52 ᶜ
ZnO	4.51 ± 0.07 ᵇ	25.89 ± 0.75 ᶜ	0.01 ± 0.01 ᵃ	0.24 ± 0.06 ᵇ	26.14 ± 0.76 ᶜ
Fe_2_O_3_	3.79 ± 0.05 ᶜ	35.09 ± 1.02 ᵃᵇ	0.02 ± 0.01 ᵃ	0.57 ± 0.09 ᵃ	35.68 ± 1.03 ᵃ

^1^ Values are the means ± SEM, n = 5. ^a–c^ Treatment groups not indicated by the same letter in the same column are significantly different according to one-way ANOVA with post hoc Duncan test (*p* < 0.05). NI = non-injected.

**Table 7:** Effects of intraamniotic administration of experimental food-grade NP solutions on crypt goblet cell diameter, goblet cell count, and goblet cell type ^1^.

Treatment Group	Crypt Goblet Cell Diameter (µm)	Crypt Goblet Cell Number (Unit)			
		Acidic	Neutral	Mixture	Total
NI	2.98 ± 0.06 ᵇᶜ	8.30 ± 0.25 ᵃ	0.00 ± 0.00 ^a^	0.03 ± 0.01 ᵃ	8.33 ± 0.25 ᵃ
H_2_O	3.32 ± 0.07 ᵃ	7.41 ± 0.22 ᵇ	0.00 ± 0.00 ^a^	0.03 ± 0.01 ᵃ	7.44 ± 0.22 ᵇ
TiO_2_	2.75 ± 0.05 ᵈ	7.29 ± 0.24 ᵇ	0.00 ± 0.00 ^a^	0.00 ± 0.00 ᵇ	7.29 ± 0.24 ᵇ
SiO_2_	2.96 ± 0.08 ᵇᶜ	7.89 ± 0.26 ᵃᵇ	0.00 ± 0.00 ^a^	0.00 ± 0.00 ᵇ	7.89 ± 0.26 ᵃᵇ
ZnO	3.04 ± 0.07 ᵇ	7.59 ± 0.22 ᵇ	0.00 ± 0.00 ^a^	0.00 ± 0.00 ᵇ	7.59 ± 0.22 ᵇ
Fe_2_O_3_	2.79 ± 0.07 ᶜᵈ	8.42 ± 0.26 ᵃ	0.00 ± 0.00 ^a^	0.00 ± 0.00 ᵇ	8.42 ± 0.26 ᵃ

^1^ Values are the means ± SEM, n = 5. ^a–d^ Treatment groups not indicated by the same letter in the same column are significantly different according to one-way ANOVA with post hoc Duncan test (*p* < 0.05). NI = non-injected.

**Table 8:** Effects of intraamniotic administration of experimental food-grade NP solutions on Paneth cells per crypt and Paneth cells diameter ^1^.

Treatment Group	Number of Paneth Cells per Crypt	Crypt Paneth Cell Diameter (µm)
NI	2.24 ± 0.08 ᵈ	1.56 ± 0.03 ᵈ
H_2_O	1.89 ± 0.07 ᵉ	1.89 ± 0.05 ᶜ
TiO_2_	2.91 ± 0.09 ᵃ	2.79 ± 0.05 ᵃ
SiO_2_	2.54 ± 0.09 ᶜ	2.18 ± 0.05 ᵇ
ZnO	2.82 ± 0.09 ᵃᵇ	2.90 ± 0.05 ᵃ
Fe_2_O_3_	2.61 ± 0.08 ᵇᶜ	1.56 ± 0.03 ᵈ

^1^ Values are the means ± SEM, n = 5. ^a–e^ Treatment groups not indicated by the same letter in the same column are significantly different according to one-way ANOVA with post hoc Duncan test (*p* < 0.05). NI = non-injected.

## Data Availability

Data are available upon reasonable request.

## Supplementary Materials

The following supporting information can be downloaded at: https://www.mdpi.com/article/10.3390/antiox12020431/s1, Table S1. Delivered Power of the BRANSON Sonifier^®^ SFX550 according to a chosen setting or amplitude (%); Figure S1. Critical DSE (J/mL) determination using TiO_2_ NP as a reference NP. (A) TiO_2_ NP hydrodynamic size measured by DLS. (B) Size fold change (%) relative to the previous measurement. (C) Table listing the delivered sonication energy (J/mL) along the sonication time (seconds) to achieve the lowest particle agglomeration state (nm).

## References

[1] Bergin I.L., Witzmann F.A. (2013). Nanoparticle toxicity by the gastrointestinal route: Evidence and knowledge gaps. Int. J. Biomed. Nanosci. Nanotechnol..

[2] Jeevanandam J., Barhoum A., Chan Y.S., Dufresne A., Danquah M.K. (2018). Review on nanoparticles and nanostructured materials: History, sources, toxicity and regulations. Beilstein J. Nanotechnol..

[3] McClements D.J., Xiao H. (2017). Is nano safe in foods? Establishing the factors impacting the gastrointestinal fate and toxicity of organic and inorganic food-grade nanoparticles. NPJ Sci. Food.

[4] Vassal M., Rebelo S., Pereira M.D.L. (2021). Metal oxide nanoparticles: Evidence of adverse effects on the male reproductive system. Int. J. Mol. Sci..

[5] Kumari A., Chauhan A.K. (2022). Iron nanoparticles as a promising compound for food fortification in iron deficiency anemia: A review. J. Food Sci. Technol..

[6] Oh H.J., Park Y.J., Cho J.H., Song M.H., Gu B.H., Yun W., Lee J.H., An J.S., Kim Y.J., Lee J.S. (2021). Changes in diarrhea score, nutrient digestibility, zinc utilization, intestinal immune profiles, and fecal microbiome in weaned piglets by different forms of zinc. Animals.

[7] Garcia-Rodriguez A., Moreno-Olivas F., Marcos R., Tako E., Marques C.N.H., Mahler G.J. (2020). The role of metal oxide nanoparticles, *Escherichia coli*, and *Lactobacillus rhamnosus* on small intestinal enzyme activity. Environ. Sci. Nano.

[8] Peters R.J.B., Bouwmeester H., Gottardo S., Amenta V., Arena M., Brandhoff P., Marvin H.J.P., Mech A., Moniz F.B., Pesudo L.Q. (2016). Nanomaterials for products and application in agriculture, feed and food. Trends Food Sci. Technol..

[9] Chaudhry Q., Scotter M., Blackburn J., Ross B., Boxall A., Castle L., Aitken R., Watkins R. (2008). Applications and implications of nanotechnologies for the food sector. Food Addit. Contam. Part A Chem. Anal. Control Expo. Risk Assess..

[10] Giust D., Lucío M.I., El-Sagheer A.H., Brown T., Williams L.E., Muskens O.L., Kanaras A.G. (2018). Graphene oxide–upconversion nanoparticle based portable sensors for assessing nutritional deficiencies in crops. ACS Nano.

[11] Kusi J., Scheuerman P.R., Maier K.J. (2020). Antimicrobial properties of silver nanoparticles may interfere with fecal indicator bacteria detection in pathogen impaired streams. Environ. Pollut..

[12] Voss L., Hsiao I.L., Ebisch M., Vidmar J., Dreiack N., Böhmert L., Stock V., Braeuning A., Loeschner K., Laux P. (2020). The presence of iron oxide nanoparticles in the food pigment E172. Food Chem..

[13] Dekkers S., Krystek P., Peters R.J.B., Lankveld D.P.K., Bokkers B.G.H., van Hoeven-Arentzen P.H., Bouwmeester H., Oomen A.G. (2011). Presence and risks of nanosilica in food products. Nanotoxicology.

[14] Moreno-Olivas F., Tako E., Mahler G.J. (2019). ZnO nanoparticles affect nutrient transport in an in vitro model of the small intestine. Food Chem. Toxicol..

[15] Weir A., Westerhoff P., Fabricius L., Hristovski K., von Goetz N. (2012). Titanium dioxide nanoparticles in food and personal care products. Environ. Sci. Technol..

[16] Peters R., Kramer E., Oomen A.G., Rivera Z.E., Oegema G., Tromp P.C., Fokkink R., Rietveld A., Marvin H.J., Weigel S. (2012). Presence of nano-sized silica during in vitro digestion of foods containing silica as a food additive. ACS Nano.

[17] Zhang H., Li D., Liu L., Xu L., Zhu M., He X., Liu Y. (2019). Cellular composition and differentiation signaling in chicken small intestinal epithelium. Animals.

[18] Birchenough G.M., Johansson M.E., Gustafsson J.K., Bergstrom J.H., Hansson G.C. (2015). New developments in goblet cell mucus secretion and function. Mucosal Immunol..

[19] Lamas B., Martins Breyner N., Houdeau E. (2020). Impacts of foodborne inorganic nanoparticles on the gut microbiota-immune axis: Potential consequences for host health. Part. Fibre Toxicol..

[20] Reed S., Neuman H., Glahn R.P., Koren O., Tako E. (2017). Characterizing the gut (*Gallus gallus*) microbiota following the consumption of an iron biofortified Rwandan cream seeded carioca (*Phaseolus Vulgaris* L.) bean-based diet. PLoS ONE.

[21] Zhang Y.-J., Li S., Gan R.-Y., Zhou T., Xu D.-P., Li H.-B. (2015). Impacts of gut bacteria on human health and diseases. Int. J. Mol. Sci..

[22] Natividad J.M., Verdu E.F. (2013). Modulation of intestinal barrier by intestinal microbiota: Pathological and therapeutic implications. Pharmacol. Res..

[23] Rooks M.G., Garrett W.S. (2016). Gut microbiota, metabolites and host immunity. Nat. Rev. Immunol..

[24] Huang Y.-W., Cambre M., Lee H.-J. (2017). The toxicity of nanoparticles depends on multiple molecular and physicochemical mechanisms. Int. J. Mol. Sci..

[25] Ghebretatios M., Schaly S., Prakash S. (2021). Nanoparticles in the food industry and their impact on human gut microbiome and diseases. Int. J. Mol. Sci..

[26] Gangadoo S., Nguyen H., Rajapaksha P., Zreiqat H., Latham K., Cozzolino D., Chapman J., Truong V.K. (2021). Inorganic nanoparticles as food additives and their influence on the human gut microbiota. Environ. Sci. Nano..

[27] Murugadoss S., Lison D., Godderis L., Van Den Brule S., Mast J., Brassinne F., Sebaihi N., Hoet P.H. (2017). Toxicology of silica nanoparticles: An update. Arch. Toxicol..

[28] Proquin H., Rodríguez-Ibarra C., Moonen C.G., Urrutia Ortega I.M., Briedé J.J., de Kok T.M., van Loveren H., Chirino Y.I. (2017). Titanium dioxide food additive (E171) induces ROS formation and genotoxicity: Contribution of micro and nano-sized fractions. Mutagenesis.

[29] Singh S. (2019). Zinc oxide nanoparticles impacts: Cytotoxicity, genotoxicity, developmental toxicity, and neurotoxicity. Toxicol. Mech. Method.

[30] Donaldson K., Poland C.A., Schins R.P. (2010). Possible genotoxic mechanisms of nanoparticles: Criteria for improved test strategies. Nanotoxicology.

[31] García-Rodríguez A., Vila L., Cortés C., Hernández A., Marcos R. (2018). Effects of differently shaped TiO2NPs (nanospheres, nanorods and nanowires) on the in vitro model (Caco-2/HT29) of the intestinal barrier. Part. Fibre Toxicol..

[32] Xu K., Basu N., George S. (2021). Dietary nanoparticles compromise epithelial integrity and enhance translocation and antigenicity of milk proteins: An in vitro investigation. NanoImpact.

[33] Sirelkhatim A., Mahmud S., Seeni A., Kaus N.H.M., Ann L.C., Bakhori S.K.M., Hasan H., Mohamad D. (2015). Review on zinc oxide nanoparticles: Antibacterial activity and toxicity mechanism. Nanomicro. Lett..

[34] Guo Z., Martucci N.J., Liu Y., Yoo E., Tako E., Mahler G.J. (2018). Silicon dioxide nanoparticle exposure affects small intestine function in an in vitro model. Nanotoxicology.

[35] Guo Z., Martucci N.J., Moreno-Olivas F., Tako E., Mahler G.J. (2017). Titanium dioxide nanoparticle ingestion alters nutrient absorption in an in vitro model of the small intestine. NanoImpact.

[36] Limage R., Tako E., Kolba N., Guo Z., Garcia-Rodriguez A., Marques C.N.H., Mahler G.J. (2020). TiO_2_ Nanoparticles and commensal bacteria alter mucus layer thickness and composition in a gastrointestinal tract model. Small.

[37] Tako E., Glahn R.P. (2011). Iron status of the late term broiler (*Gallus gallus*) Embryo and Hatchling. Int. J. Poult. Sci..

[38] Hou T., Tako E. (2018). The In Ovo Feeding Administration (*Gallus Gallus*)-An Emerging In vivo Approach to Assess Bioactive Compounds with Potential Nutritional Benefits. Nutrients.

[39] Mahler G.J., Esch M.B., Tako E., Southard T.L., Archer S.D., Glahn R.P., Shuler M.L. (2012). Oral exposure to polystyrene nanoparticles affects iron absorption. Nat. Nanotechnol..

[40] Kolba N., Guo Z., Olivas F.M., Mahler G.J., Tako E. (2020). Intra-amniotic administration (*Gallus gallus*) of TiO_2_, SiO_2_, and ZnO nanoparticles affect brush border membrane functionality and alters gut microflora populations. Food Chem. Toxicol..

[41] Reed S., Qin X., Ran-Ressler R., Brenna J.T., Glahn R.P., Tako E. (2014). Dietary zinc deficiency affects blood linoleic acid: Dihomo-gamma-linolenic acid (LA:DGLA) ratio; a sensitive physiological marker of zinc status in vivo (*Gallus gallus*). Nutrients.

[42] Dias D.M., Kolba N., Hart J.J., Ma M., Sha S.T., Lakshmanan N., Nutti M.R., Martino H.S.D., Glahn R.P., Tako E. (2019). Soluble extracts from carioca beans (*Phaseolus vulgaris* L.) affect the gut microbiota and iron related brush border membrane protein expression in vivo (*Gallus gallus*). Food Res. Int..

[43] International Chicken Genome Sequencing Consortium (2004). Sequence and comparative analysis of the chicken genome provide unique perspectives on vertebrate evolution. Nature.

[44] Zhu X.Y., Zhong T., Pandya Y., Joerger R.D. (2002). 16S rRNA-based analysis of microbiota from the cecum of broiler chickens. Appl Environ Microbiol.

[45] Yegani M., Korver D.R. (2008). Factors affecting intestinal health in poultry. Poult. Sci..

[46] Taurozzi J.S., Hackley V.A., Wiesner M.R. (2011). Ultrasonic dispersion of nanoparticles for environmental, health and safety assessment--issues and recommendations. Nanotoxicology.

[47] Organisation for Economic Co-operation and Development (2010). Preliminary Guidance Notes on Sample Preparation and Dosimetry for the Safety Testing of Manufactured Nanomaterials.

[48] Lomer M.C., Hutchinson C., Volkert S., Greenfield S.M., Catterall A., Thompson R.P., Powell J.J. (2004). Dietary sources of inorganic microparticles and their intake in healthy subjects and patients with Crohn’s disease. Br. J. Nutr..

[49] EFSA Panel on Food Additives and Nutrient Sources Added to Food (ANS) (2015). Scientific Opinion on the re-evaluation of iron oxides and hydroxides (E 172) as food additives. EFSA J..

[50] Decuypere E., Michels H. (1992). Incubation temperature as a management tool: A review. World’s Poult. Sci. Assoc..

[51] Tako E., Ferket P.R., Uni Z. (2005). Changes in chicken intestinal zinc exporter mRNA expression and small intestinal functionality following intra-amniotic zinc-methionine administration. J. Nutr. Biochem..

[52] Hou T., Kolba N., Glahn R.P., Tako E. (2017). Intra-Amniotic Administration (*Gallus gallus*) of Cicer arietinum and Lens culinaris Prebiotics Extracts and Duck Egg White Peptides Affects Calcium Status and Intestinal Functionality. Nutrients.

[53] Pereira da Silva B., Kolba N., Stampini Duarte Martino H., Hart J., Tako E. (2019). Soluble Extracts from Chia Seed (Salvia hispanica L.) Affect Brush Border Membrane Functionality, Morphology and Intestinal Bacterial Populations In vivo (*Gallus gallus*). Nutrients.

[54] Romanoff A.L. (1960). The Avian Embryo. Structural and Functional Development.

[55] Romanoff A.L., Romanoff A.J. (1967). Biochemistry of the Avian Embryo.

[56] Carboni J., Reed S., Kolba N., Eshel A., Koren O., Tako E. (2020). Alterations in the Intestinal Morphology, Gut Microbiota, and Trace Mineral Status Following Intra-Amniotic Administration (*Gallus gallus*) of Teff (Eragrostis tef) Seed Extracts. Nutrients.

[57] Pacifici S., Song J., Zhang C., Wang Q., Glahn R.P., Kolba N., Tako E. (2017). Intra Amniotic Administration of Raffinose and Stachyose Affects the Intestinal Brush Border Functionality and Alters Gut Microflora Populations. Nutrients.

[58] Tako E., Glahn R.P., Knez M., Stangoulis J.C.R. (2014). The effect of wheat prebiotics on the gut bacterial population and iron status of iron deficient broiler chickens. Nutr. J..

[59] Martino H.S.D., Kolba N., Tako E. (2020). Yacon (*Smallanthus sonchifolius*) flour soluble extract improve intestinal bacterial populations, brush border membrane functionality and morphology in vivo (*Gallus gallus*). Food Res. Int..

[60] Warkentin T., Kolba N., Tako E. (2020). Low Phytate Peas (*Pisum sativum* L.) Improve Iron Status, Gut Microbiome, and Brush Border Membrane Functionality In vivo (*Gallus gallus*). Nutrients.

[61] Dreiling C., Brown D., Casale L., Kelly L. (1987). Muscle Glycogen: Comparison of Iodine Binding and Enzyme Digestion Assays and Application to Meat Samples. Meat Sci..

[62] Hartono K., Reed S., Ankrah N.A., Glahn R.P., Tako E. (2015). Alterations in gut microflora populations and brush border functionality following intra-amniotic daidzein administration. RSC Adv..

[63] Cheng J., Kolba N., Sisser P., Turjeman S., Even C., Koren O., Tako E. (2022). Intraamniotic Administration (*Gallus gallus*) of Genistein Alters Mineral Transport, Intestinal Morphology, and Gut Microbiota. Nutrients.

[64] Keller A.A., Wang H., Zhou D., Lenihan H.S., Cherr G., Cardinale B.J., Miller R., Ji Z. (2010). Stability and aggregation of metal oxide nanoparticles in natural aqueous matrices. Environ. Sci. Technol..

[65] Pareek V., Bhargava A., Bhanot V., Gupta R., Jain N., Panwar J. (2018). Formation and Characterization of Protein Corona around Nanoparticles: A Review. J. Nanosci. Nanotechnol..

[66] Sohal I.S., Cho Y.K., O’Fallon K.S., Gaines P., Demokritou P., Bello D. (2018). Dissolution Behavior and Biodurability of Ingested Engineered Nanomaterials in the Gastrointestinal Environment. ACS Nano.

[67] Hartmann N.B., Jensen K.A., Baun A., Rasmussen K., Rauscher H., Tantra R., Cupi D., Gilliland D., Pianella F., Riego Sintes J.M. (2015). Techniques and Protocols for Dispersing Nanoparticle Powders in Aqueous Media-Is there a Rationale for Harmonization?. J. Toxicol. Environ. Health B Crit. Rev..

[68] Jensen K., Kembouche Y., Christiansen E., Jacobsen N., Wallin H. (2014). The Generic NANOGENOTOX Dispersion Protocol—Standard Operation Procedure (SOP).

[69] Zhang Y., Chen Y., Westerhoff P., Hristovski K., Crittenden J.C. (2008). Stability of commercial metal oxide nanoparticles in water. Water Res..

[70] French R.A., Jacobson A.R., Kim B., Isley S.L., Penn R.L., Baveye P.C. (2009). Influence of Ionic Strength, pH, and Cation Valence on Aggregation Kinetics of Titanium Dioxide Nanoparticles. Environ. Sci. Tech..

[71] Jeong J., Eide D.J. (2013). The SLC39 family of zinc transporters. Mol. Asp. Med..

[72] Maares M., Haase H. (2020). A Guide to Human Zinc Absorption: General Overview and Recent Advances of In Vitro Intestinal Models. Nutrients.

[73] Kambe T., Hashimoto A., Fujimoto S. (2014). Current understanding of ZIP and ZnT zinc transporters in human health and diseases. Cell Mol. Life. Sci..

[74] Kimura T., Kambe T. (2016). The Functions of Metallothionein and ZIP and ZnT Transporters: An Overview and Perspective. Int. J. Mol. Sci..

[75] Huang L., Tepaamorndech S. (2013). The SLC30 family of zinc transporters—A review of current understanding of their biological and pathophysiological roles. Mol. Asp. Med..

[76] Ghareeb K., Awad W.A., Soodoi C., Sasgary S., Strasser A., Böhm J. (2013). Effects of Feed Contaminant Deoxynivalenol on Plasma Cytokines and mRNA Expression of Immune Genes in the Intestine of Broiler Chickens. PLoS ONE.

[77] Gutsmann T., Schromm A.B., Brandenburg K. (2007). The physicochemistry of endotoxins in relation to bioactivity. Int. J. Med. Microbiol..

[78] Hayden M.S., Ghosh S. (2014). Regulation of NF-kappaB by TNF family cytokines. Semin. Immunol..

[79] Vasto S., Mocchegiani E., Malavolta M., Cuppari I., Listi F., Nuzzo D., Ditta V., Candore G., Caruso C. (2007). Zinc and inflammatory/immune response in aging. Ann. N. Y. Acad. Sci..

[80] Jarosz M., Olbert M., Wyszogrodzka G., Młyniec K., Librowski T. (2017). Antioxidant and anti-inflammatory effects of zinc. Zinc-dependent NF-κB signaling. Inflammopharmacology.

[81] Uni Z., Ferket P.R., Tako E., Kedar O. (2005). In ovo feeding improves energy status of late-term chicken embryos. Poult. Sci..

[82] Mercier-Bonin M., Despax B., Raynaud P., Houdeau E., Thomas M. (2018). Mucus and microbiota as emerging players in gut nanotoxicology: The example of dietary silver and titanium dioxide nanoparticles. Crit. Rev. Food. Sci. Nutr..

[83] Fan Y.K., Croom J., Christensen V.L., Black B.L., Bird A.R., Daniel L.R., McBride B.W., Eisen E.J. (1997). Jejunal glucose uptake and oxygen consumption in turkey poults selected for rapid growth. Poult. Sci..

[84] Xu Z.R., Hu C.H., Xia M.S., Zhan X.A., Wang M.Q. (2003). Effects of dietary fructooligosaccharide on digestive enzyme activities, intestinal microflora and morphology of male broilers. Poult. Sci..

[85] Khajeh Bami M., Afsharmanesh M., Ebrahimnejad H. (2020). Effect of Dietary Bacillus coagulans and Different Forms of Zinc on Performance, Intestinal Microbiota, Carcass and Meat Quality of Broiler Chickens. Probiotics Antimicrob. Proteins.

[86] Nikolenko V.N., Oganesyan M.V., Sankova M.V., Bulygin K.V., Vovkogon A.D., Rizaeva N.A., Sinelnikov M.Y. (2021). Paneth cells: Maintaining dynamic microbiome-host homeostasis, protecting against inflammation and cancer. Bioessays.

[87] Kim Y.S., Ho S.B. (2010). Intestinal goblet cells and mucins in health and disease: Recent insights and progress. Curr. Gastroenterol. Rep..

[88] Wang X., Kolba N., Liang J., Tako E. (2019). Alterations in gut microflora populations and brush border functionality following intra-amniotic administration (Gallus gallus) of wheat bran prebiotic extracts. Food Funct..

[89] Kim J.J., Khan W.I. (2013). Goblet cells and mucins: Role in innate defense in enteric infections. Pathogens.

[90] Qiu K., Durham P.G., Anselmo A.C. (2018). Inorganic nanoparticles and the microbiome. Nano. Research.

[91] Engevik M.A., Luk B., Chang-Graham A.L., Hall A., Herrmann B., Ruan W., Endres B.T., Shi Z., Garey K.W., Hyser J.M. (2019). Bifidobacterium dentium Fortifies the Intestinal Mucus Layer via Autophagy and Calcium Signaling Pathways. mBio.

[92] O’Callaghan A., van Sinderen D. (2016). Bifidobacteria and Their Role as Members of the Human Gut Microbiota. Front. Microbiol..

[93] Sánchez B., Champomier-Vergès M.-C., Collado M.d.C., Anglade P., Baraige F., Sanz Y., de los Reyes-Gavilán C.G., Margolles A., Zagorec M. (2007). Low-pH adaptation and the acid tolerance response of Bifidobacterium longum biotype longum. Appl. Environ. Microbiol..

[94] Seiler C., Berendonk T.U. (2012). Heavy metal driven co-selection of antibiotic resistance in soil and water bodies impacted by agriculture and aquaculture. Front. Microbiol..

[95] Cao X., Han Y., Gu M., Du H., Song M., Zhu X., Ma G., Pan C., Wang W., Zhao E. (2020). Foodborne Titanium Dioxide Nanoparticles Induce Stronger Adverse Effects in Obese Mice than Non-Obese Mice: Gut Microbiota Dysbiosis, Colonic Inflammation, and Proteome Alterations. Small.

[96] Rinninella E., Cintoni M., Raoul P., Mora V., Gasbarrini A., Mele M.C. (2021). Impact of Food Additive Titanium Dioxide on Gut Microbiota Composition, Microbiota-Associated Functions, and Gut Barrier: A Systematic Review of In vivo Animal Studies. Int. J. Environ. Res. Public Health.

