# Supplementary material for: Food-Grade Metal Oxide Nanoparticles Exposure Alters Intestinal Microbial Populations, Brush Border Membrane Functionality and Morphology, In Vivo (Gallus gallus)
Source: Antioxidants (Basel). 2023 Feb 9;12(2):431. doi: 10.3390/antiox12020431 (PMC9952780; doi:10.3390/antiox12020431)
Supplement: Supplementary file 1 [file antioxidants-12-00431-s001.zip › antioxidants-2201988-supplementary.pdf]

antioxidants

Supplementary Materials: Food-Grade Metal Oxide Nanoparticles Exposure Alters Intestinal Microbial Populations, Brush Border Membrane Functionality and Morphology, In Vivo (*Gallus gallus*)

Jacquelyn Cheng [1], Nikolai Kolba [1], Alba García-Rodríguez [2,3], Cláudia N.H. Marques [3], Gretchen J. Mahler [2] and Elad Tako [1,*]

[1] Department of Food Science, Cornell University, 411 Tower Road, Ithaca, NY 14853, USA
[2] Department of Biomedical Engineering, Binghamton University, 4400 Vestal Parkway East, Binghamton, NY 13902, USA
[3] Department of Biological Sciences, Binghamton University, 4400 Vestal Parkway East, Binghamton, NY 13902, USA
* Correspondence: et79@cornell.edu; Tel.: +1-607-255-0884

1. Sonicator Calibration and Critical Delivered Sonication Energy Determination

The calorimetric method has been previously proposed to standardize sonochemistry studies and calculate the amount of acoustic energy delivered to a liquid medium subject to direct sonication. Briefly, at a given sonicator setting (amplitude in %), the temperature in the liquid is recorded over time, and the effective delivered power can then be determined using the following equation:

$$P = \frac{dT}{dt} M C_p \quad (1)$$

Where P is the delivered acoustic power (W), T and t are temperature (K) and time (s), respectively, Cp is the specific heat of the liquid (J g-1 K-1) and M is the mass of the liquid (g). The obtained results are listed in Table S1.

The critical DSE of an engineered NP is defined as the delivered sonication energy (in J/mL) required to achieve a monodisperse solution at the lowest particle agglomeration state in DI H2O. The ultimate goal of the optimization procedure was to achieve the desired degree of particle dispersion with the least possible energy input, in order to minimize unwanted side effects [1]. Therefore, all dispersions for all four NP were analyzed for hydrodynamic size by DLS every 30 s when sonicating at 10% of amplitude (Table S1) The final sonication time was chosen according to the first minimum difference in NP deagglomeration among two consecutive measurements (Figure S1).

Table S1. Delivered Power of the BRANSON Sonifier® SFX550 according to a chosen setting or amplitude (%).

Amplitude (%)	Delivered Power (W)
10 %	7.3255
15 %	10.6743
20 %	15.2789
25 %	19.0463
30 %	25.5346

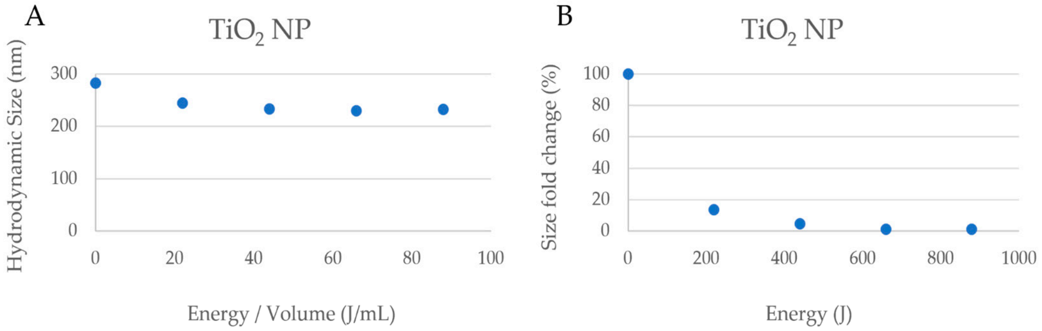

C

Time (s)	Total Energy Delivered (Joules)	Energy / Volume (Joules/mL)	Hydrodynamic Size (nm)	% of Size Difference
0	0	0	282.26	100
30	219.76	21.97	244.06	13.53
60	439.53	43.95	232.76	4.62
90	659.29	65.92	229.7	1.31
120	879.06	87.90	232.26	1.11

Figure S1. Critical DSE (J/mL) determination using TiO2 NP as a reference NP. (A) TiO2 NP hydrodynamic size measured by DLS. (B) Size fold change (%) relative to the previous measurement. (C) Table listing the delivered sonication energy (J/mL) along the sonication time (seconds) to achieve the lowest particle agglomeration state (nm).

3. Glycogen Concentration Analysis as Measurement of Energetic Status

Glycogen content analysis of the pectoralis muscle and liver was performed as previously described [2–5]. The frozen pectoral muscle or frozen liver samples were homogenized in 8% perchloric acid. Pectoral muscle samples were then centrifuged at 12,000 x g for 15 min, and liver samples were centrifuged at 4000 x g for 15 min at 4°C. The supernatant was discarded, and 1 mL of petroleum ether was added to each tube. After mixing, the petroleum ether fraction was discarded and samples from the bottom layer were transferred to a 96-well plate. 300 µL of iodine reagent was added and the reaction was carried out for 10 min. All samples were read at absorbance 450 nm in a spectrophotometer (Epoch, BioTek, VT, USA) and the glycogen content was calculated according to a standard curve where the glycogen content present in each sample was determined by multiplying the weight of the tissue by the amount of glycogen per 1 g of wet tissue.

4. Total RNA Isolation and Real Time Polymerase Chain Reaction (RT-qPCR)

Total RNA was extracted from 30 mg of duodenal tissue using a Qiagen RNeasy Mini Kit (Qiagen Inc., Germantown, MD) according to the manufacturer's protocol under RNase free conditions. Total RNA was eluted in 50 µL of RNase-free water. RNA was quantified with a NanoDrop 2000 (ThermoFisher Scientific, Waltham, MA) at A260/280. RNA was stored at -80˚C until use.

The primers used in the real-time polymerase chain reactions (RT-PCR) were designed using Real-Time Primer Design Tool software (IDT DNA, Coralvilla, IA) based on 11 gene sequences from the GenBank database. The sequences are shown in the manuscript in Table 2. The amplicon length was limited to 90 to 150 bp, the length of the primers was from 17-to 25-mer, and the GC content was between 41 and 55%. The specificity of the primers was tested by performing a BLAST search against the genomic NCBI database.

Reverse-transcription to cDNA was done using the ImProm-II Reverse Transcriptase Kit (Promega Corporation, Madison, WI) based on manufacturer's instructions using a 20 µL reverse transcriptase reaction. The reverse transcriptase reaction consisted of 1 µg total

RNA template, 10 μM random hexamer primers, and 2 mM of oligo-dT primers. All reactions were performed under the following conditions: 94˚C for 5 min, 60 min at 42˚C, 70˚C for 15 min, and hold at 4˚C. The concentration of cDNA obtained was determined with a NanoDrop 2000 at A260/280 with an extinction coefficient of 33 for single stranded DNA.

RT-PCR was performed with a Bio-RadCFX96 Touch (Hercules, CA, USA). The 10 μL RT-PCR mixtures consisted of cDNA (2 μg), 2X BioRad SSO Advanced Universal SYBR Green Supermix (Cat #1725274, Hercules, CA, USA), forward and reverse primers, and nuclease-free water (for the no template control). The no template control of nuclease-free water was included to exclude DNA contamination in the PCR mix. All reactions were performed in duplicates and under the following conditions: initial denaturing at 95˚C for 30 s, 40 cycles of denaturing at 95˚C for 15 s, various annealing temperatures according to IDT for 30 s and elongating at 60˚C for 30 s. After the cycling process was completed, melting curves was determined from 65°C to 95°C with increments of 0.5°C for 5 s to ensure amplification of a single product. RT-PCR efficiency values for the eleven genes were as follows: DcytB, 1.046; DMT1, 0.998; Ferroportin, 1.109; ZIP1, 0.921; ZnT1, 1.09; SGLT1, 0.994; SI, 1.032; MUC2, 1.022; NK-κβ, 1.113; TNF-α, 1.046; IL-8, 0.998; 18s rRNA, 0.994. Gene expression levels were obtained from Ct values based on the 'second derivative maximum' as computed by the Bio-Rad CFX Maestro Software (Bio-Rad, Hercules, CA, USA). Gene expression was normalized to the expression of 18S.

5. Microbial Sample collection, Cecal Content DNA Isolation, Polymerase Chain Reaction Amplification of 16s rDNA

As was previously described [6–8]. Cecal contents (200 mg) were placed into a sterile 15 mL tube (Corning, Corning, NY, USA) containing 9 mL of sterile 1X phosphate buffered saline and vortexed with 3 mm silicone beads for 3 min to homogenize. Samples were centrifuged at 1000 x g for 1 min, the supernatant collected, and centrifuged at 12,000 x g for 5 min. The resulting pellet was washed twice with 1X phosphate buffered saline. For DNA extraction, the pellet was resuspended in 50 mM EDTA and was incubated with 10 mg/mL lysozyme (Sigma Aldrich CO., St. Louis, MO) for 45 min at 37°C. The bacterial genomic DNA was then isolated with the Wizard Genomic DNA purification kit (Promega Corp., Madison, WI) per the manufacturer's instructions. Lactobacillus spp., Bifidobacterium spp., Clostridium spp., and E. coli primers were designed according to previously published data [9]. Universal primers for the invariant region of bacterial 16S rRNA were utilized for results normalization. PCR products were separated via electrophoresis using a 2% agarose gel, stained with ethidium bromide, and measured using Quantity One 1-D analysis software (Bio-Rad, Hercules, CA).

References

1. Taurozzi, J.S.; Hackley, V.A.; Wiesner, M.R. Ultrasonic dispersion of nanoparticles for environmental, health and safety assessment--issues and recommendations. Nanotoxicology 2011, 5, 711-729, doi:10.3109/17435390.2010.528846.
2. Pereira da Silva, B.; Kolba, N.; Stampini Duarte Martino, H.; Hart, J.; Tako, E. Soluble Extracts from Chia Seed (Salvia hispanica L.) Affect Brush Border Membrane Functionality, Morphology and Intestinal Bacterial Populations In Vivo (Gallus gallus). Nutrients 2019, 11, doi:10.3390/nu11102457.
3. Martino, H.S.D.; Kolba, N.; Tako, E. Yacon (Smallanthus sonchifolius) flour soluble extract improve intestinal bacterial populations, brush border membrane functionality and morphology in vivo (Gallus gallus). Food Res Int 2020, 137, 109705, doi:10.1016/j.foodres.2020.109705.
4. Warkentin, T.; Kolba, N.; Tako, E. Low Phytate Peas (Pisum sativum L.) Improve Iron Status, Gut Microbiome, and Brush Border Membrane Functionality In Vivo (Gallus gallus). Nutrients 2020, 12, doi:10.3390/nu12092563.
5. Dreiling, C.; Brown, D.; Casale, L.; Kelly, L. Muscle Glycogen: Comparison of Iodine Binding and Enzyme Digestion Assays and Application to Meat Samples. Meat Science 1987, 20, 167-177.
6. Hartono, K.; Reed, S.; Ankrah, N.A.; Glahn, R.P.; Tako, E. Alterations in gut microflora populations and brush border functionality following intra-amniotic daidzein administration. RSC Advances 2015, 5, 6407-6412, doi:10.1039/c4ra10962g.

7. Kolba, N.; Guo, Z.; Olivas, F.M.; Mahler, G.J.; Tako, E. Intra-amniotic administration (Gallus gallus) of TiO2, SiO2, and ZnO nanoparticles affect brush border membrane functionality and alters gut microflora populations. Food Chem Toxicol 2020, 135, 110896, doi:10.1016/j.fct.2019.110896.
8. Dias, D.M.; Kolba, N.; Hart, J.J.; Ma, M.; Sha, S.T.; Lakshmanan, N.; Nutti, M.R.; Martino, H.S.D.; Glahn, R.P.; Tako, E. Soluble extracts from carioca beans (Phaseolus vulgaris L.) affect the gut microbiota and iron related brush border membrane protein expression in vivo (Gallus gallus). Food Res Int 2019, 123, 172-180, doi:10.1016/j.foodres.2019.04.060.
9. Zhu, X.Y.; Zhong, T.; Pandya, Y.; Joerger, R.D. 16S rRNA-based analysis of microbiota from the cecum of broiler chickens. Appl Environ Microbiol 2002, 68, 124-137, doi:10.1128/aem.68.1.124-137.2002.
